# A cross-sectional study of the role of men and the knowledge of danger signs during pregnancy in southern Mozambique

**DOI:** 10.1186/s12884-020-03265-4

**Published:** 2020-09-29

**Authors:** Anna Galle, Malica De Melo, Sally Griffin, Nafissa Osman, Kristien Roelens, Olivier Degomme

**Affiliations:** 1 International Centre for Reproductive Health, Department of Public Health and Primary Care, Ghent University, Corneel Heymanslaan 10, entrance 75, UZP 114, 9000 Ghent, Belgium; 2 International Centre for Reproductive Health - Mozambique, Rua das Flores No 34, Impasse 1085/87, Maputo, Mozambique; 3 Department of Obstetrics/Gynecology, Faculty of Medicine, Eduardo Mondlane University, Av. Salvador Allende-57, Maputo, Mozambique

## Abstract

**Background:**

The role of the male partner and wider family in maternal health, especially in case of emergencies, has been receiving increasing attention over the last decade. Qualitative research has highlighted that women depend on others to access high quality maternity care. Currently little is known about these factors in relation to maternal health in Mozambique.

**Methods:**

A cross sectional household survey was conducted with men and women in southern Mozambique about decision making, financial support and knowledge of danger signs. A multivariable logistic model was used to identify factors associated with knowledge of danger signs and Cohen’s kappa for agreement among couples.

**Results:**

A total of 775 men and women from Marracuene and Manhica districts were interviewed. Maternal health care decisions were frequently made jointly by the couple (32–49%) and financial support was mainly provided by the man (46–80%). Parental and parent-in-law involvement in decision making and financial support was minimal (0–3%). The average number of danger signs respondents knew was 2.05 and no significant difference (*p* = 0.294) was found between men and women. Communication with the partner was a significant predictor for higher knowledge of danger signs for both men (*p* = 0.01) and women (*p* = 0.03). There was very low agreement within couples regarding decision making (*p* = 0.04), financial support (*p* = 0.01) and presence at antenatal care consultations (*p* = 0.001). Results suggest women and men have a high willingness for more male participation in antenatal care, although their understanding of what constitutes this participation is not clear.

**Conclusion:**

The study findings highlight the important role men play in decision making and financial support for maternal health care issues. Strengthening male involvement in antenatal care services, by investing in counselling and receiving couples, could help accelerate gains in maternal health in Mozambique. Maternal health care studies should collect more data from men directly as men and women often report different views and behavior regarding maternal health care issues and male involvement.

## Background

Maternal mortality remains unacceptably high in most low- and middle-income countries. As a consequence, improving maternal health is still a high priority under the Sustainable Development Goals (SDGs). The SDGs agenda places greater emphasis than the Millennium Development Goals (MDGs) on cross-sectoral links across social, economic and environmental pillars [1]. Furthermore, the SDGs aim to reduce inequalities within and between countries as key mechanism to improve health for all. The global target for maternal health under the SDGs states that by 2030, the global Maternal Mortality Ratio (MMR) should be reduced to fewer than 70 maternal deaths per 100,000 live births, and that at national level no country should have a MMR greater than 140 maternal deaths per 100,000 live births [1].

In Mozambique, one of the poorest countries of Sub-Saharan Africa, the latest estimated MMR is still very high at 289 per 1,000,000 live births in 2017, with wide variations across the country [2]. As part of efforts to reduce MMR, Mozambique adopted the World Health Organization’s (WHO) focused antenatal care (FANC) program consisting of four visits for low-risk pregnancies without complications [3]. This antenatal care (ANC) program includes health promotion and the prevention, detection and treatment of diseases during pregnancy. During the MDGs era, the global coverage of ANC contacts improved in almost all low and middle income countries (LMICs), but the content and quality of antenatal care has been questioned [4, 5]. Several studies have shown that some practices from the FANC model, such as counselling on danger signs and hypertensive disease management, are often neglected by providers in LMICs [6–8]. A recent study in ten LMICs found that coverage of provision of information on complications during pregnancy was extremely low [9], despite the fact that communicating such information requires no supplies or equipment. Informing women and their partners about danger signs during pregnancy is an essential step for appropriate and timely referral of pregnant women in case of life threatening emergencies. Furthermore counselling on danger signs is also identified as a critical component of ANC by women themselves [10], motivating them to seek ANC. Investing in information, education and communication programs during pregnancy can prevent maternal mortality caused by the first delay of the three delays model of maternal mortality, which proposes that maternal mortality is associated with delays in: 1) deciding to seek care; 2) reaching the healthcare facility; and 3) receiving care [11].

WHO updated its ANC guidelines in 2018 [12] and now recommends that each woman attends eight or more routine ANC consultations during pregnancy, rather than the four visits suggested by the previous model. The new guidelines are more comprehensive than the previous model, with an increased focus on the experience of care. According to WHO, experience of quality care requires effective communication with the woman and her family, provision of care with respect and dignity, and access to social and emotional support. However, considering many countries already struggle to ensure adherence to the recommendations contained in the previous model [13], it will be even more challenging for countries with limited resources to adhere to these more comprehensive recommendations. The Ministry of Health (MOH) in Mozambique has not yet adopted the new ANC model but has begun to address quality of ANC, for example through the implementation and evaluation of a supply kit for ANC and scaling up the training of Mother and Child Health (MCH) nurses [14].

In Maputo Province (the most southerly province in Mozambique), 74% of all pregnant women complete the WHO-recommended four or more antenatal visits and 87.5% deliver in a health facility [15]. At community level persisting barriers continue to prevent timely care-seeking behavior for obstetric emergencies and delivery. A qualitative study, conducted in 2016 in southern Mozambique, revealed that unfamiliarity with danger signs, especially among partners, was a major reason for not seeking care [16]. Male partners, neighbors and mothers-in-law are key actors in the referral of pregnant women in rural southern Mozambique [16]. Although pregnancy and childbirth are traditionally considered as the women’s domain, women often do not have independent access to maternal health care services due to economic dependency and gender inequality [17]. From the existing literature from LMICs it is unclear to what extent male partners are aware of danger signs, taking the final decisions, or providing financial and logistical support to reach health care services [18–20]. The majority of maternal health care studies gather data from women only while often the wider community, including the male partner, is involved when obstetric emergencies occur [21, 22]. In order to design and implement effective and comprehensive maternal health care programs, further insight into decision making and maternal health care knowledge at community level is highly needed.

Awareness of danger signs during pregnancy among both men and women has not been previously studied in southern Mozambique. Moreover, it is unclear what role men play in terms of decision making and financial support in this setting. This study aimed to assess decision making regarding maternal health care issues, financial support for ANC and delivery, and the knowledge of danger signs of both men and women of reproductive age at community level.

## Methods

### Study setting

The study took place in Maputo province in the neighbouring districts of Marracuene and Manhiça, which had respectively 84,975 and 157,642 inhabitants in 2007 [23]. Formal maternal healthcare is provided entirely by public health services in this area, organised by a broad network of primary health care centers with secondary and tertiary referral centers [15]. At least 94% of women in Maputo Province receive one ANC during pregnancy and 87.5% of women deliver in a health facility [15]. Teenage pregnancy is very common: 25.8% of women between 15 and 19 years old have already been pregnant [15]. The most common direct causes for maternal deaths in Mozambique are hemorrhage, sepsis and eclampsia and among indirect causes HIV and malaria infections take the lead [24–26]. Compared to the rest of the country, Maputo Province has a reasonable coverage of health care centers: 90% of the population has a health care center within a 1 h drive [27].

### Study design

A cross-sectional descriptive survey was conducted between June and August 2017. The study was nested in a cohort study in which 383 households were followed over a period of 4 years (from 2014 until 2017) in Manhiça and Marracuene districts, Mozambique. All participants within the cohort study were questioned annually about family planning knowledge, attitudes and practices. Additional questions relating to the current study were included in the final round of data collection. The questionnaire can be found as an additional file [Additional file 1].

### Sample size

Families were recruited through a simple, district-stratified random sampling process with allocation proportional to size within each stratum (as shown in Table 1). According to the National Institute of Statistics, 35,454 and 20,712 households lived in Manhica and Marracuene respectively in 2007. Based on the sample size calculation (as shown in Table 1) the aim was to include 383 households, of which 242 in Manhiça and 141 in Marracuene. Considering a traditional household usually consists of at least one man and one woman of reproductive age, the aim was to include 766 men and women.

**Table 1:** Stratified Sample Technique according to Haddad et al. (2004) for calculating the sample size [28]

District	Stratum Size (N_**h**_)	Stratum Weight (W_**h**_)	Sample (n_**h**_)
Manhiça	35,454	0.63	242
Marracuene	20,712	0.37	141
**Total**	**56,166**	**1.00**	**383**

### Data collection tool

**Fig. 1:**
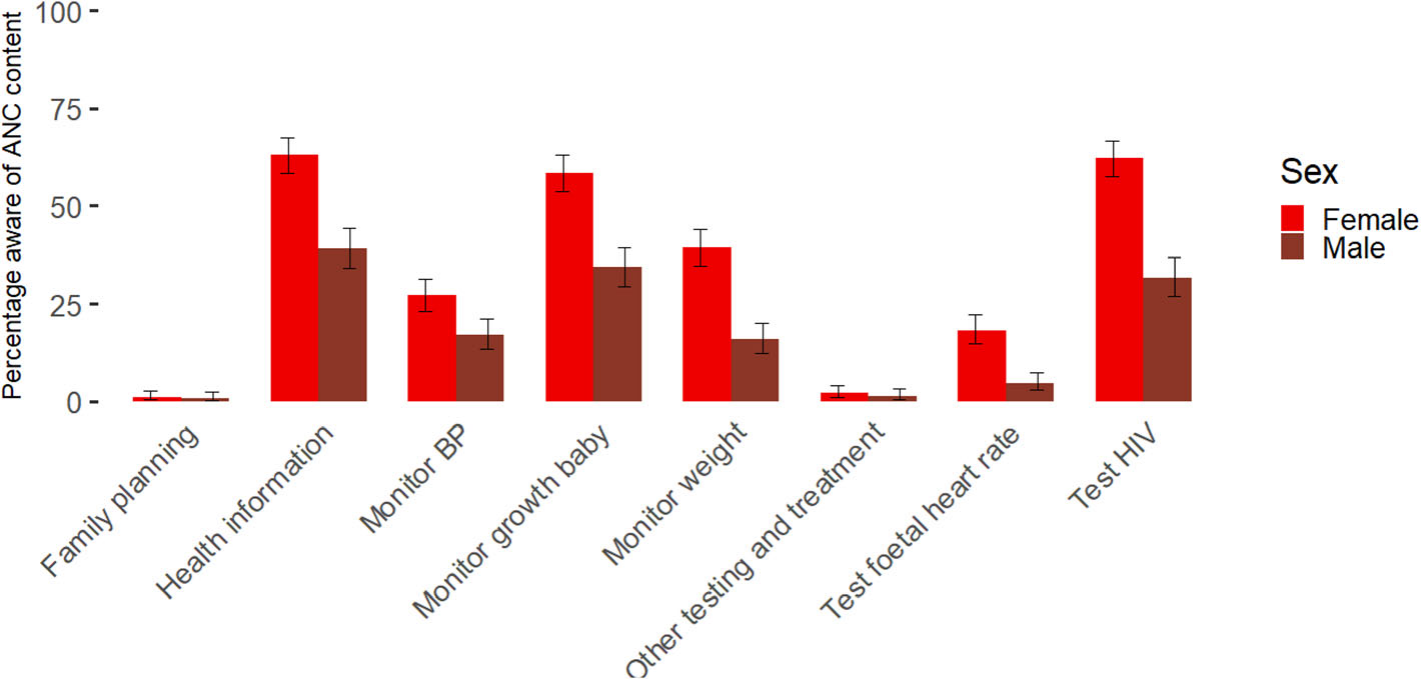
Knowledge content ANC by sex in percentage with confidence intervals for proportions

The questionnaire consisted of questions regarding sociodemographic characteristics, knowledge of content of ANC, knowledge of danger signs during pregnancy and level of male involvement during pregnancy. The knowledge of content of ANC was assessed by the open-ended question: “What happens during ANC?”. The items listed by the respondent were categorised by the interviewer under different categories. The categories (see Fig. 1) were based on the minimum package of services to be provided by antenatal care according to WHO and MoH guidelines [29, 30]. Items not fitting in these categories were noted under the option “others”. Items listed under “others” were revised by the research team and if necessary added to a certain category after data collection. Tetanus vaccination, anaemia screening and intermittent preventive treatment of malaria were categorised under “other testing and treatments”.

**Fig. 2:**
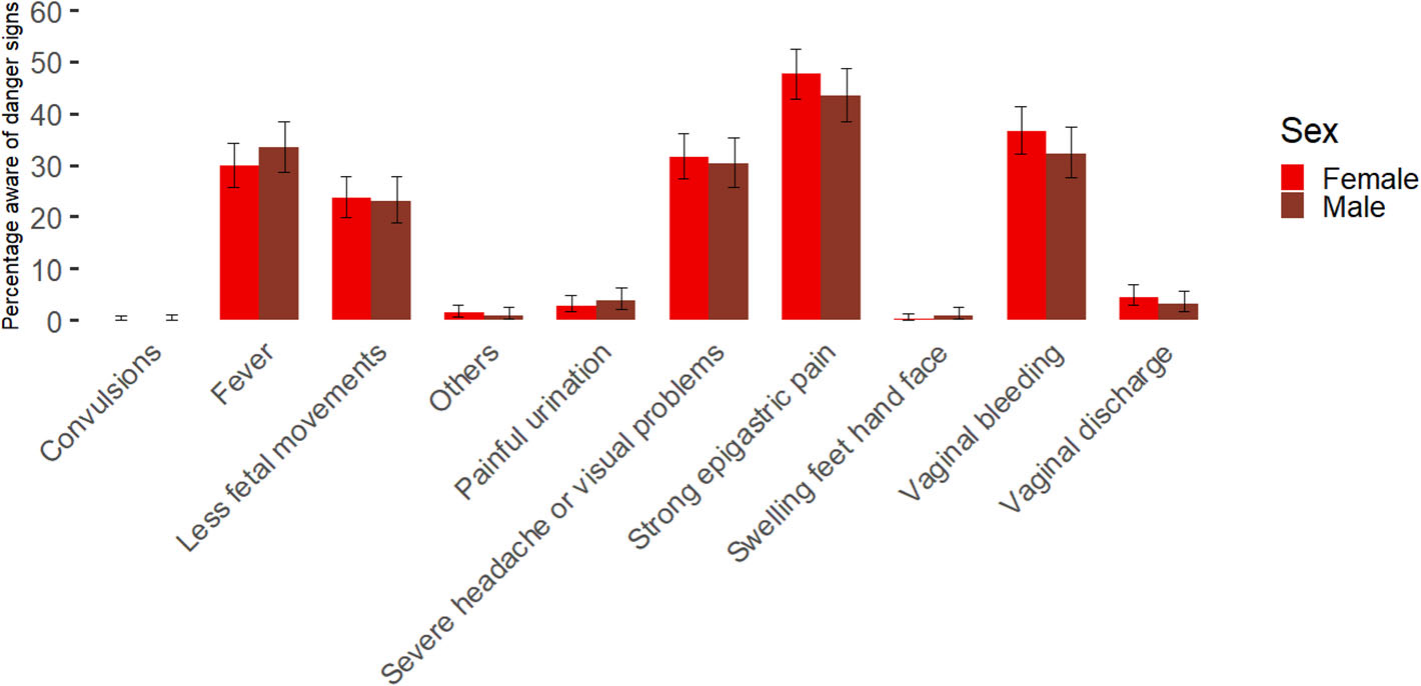
Knowledge danger signs by sex in percentage with confidence intervals for proportions

Responses regarding knowledge of danger signs was assessed by the open ended question: “What are the danger signs during pregnancy?”. Responses were categorised by the interviewer under predetermined categories or noted under “others”. Items listed under “others” were revised by the research team and if necessary added to another category after data collection. Final categories of danger signs included: 1. Vaginal bleeding 2. Convulsions or fitting 3. Severe headache and/or blurred vision 4. Fever 5. Painful urination 6. Severe abdominal/epigastric pain 7. Reduced fetal movements 8. Swelling of fingers, face, and legs 9. Abnormal vaginal discharge 10. Others (see Fig. 2). The category “others” included answers referring to a feeling of extreme weakness, weight loss or fast and difficult breathing. Abnormal vaginal discharge included responses referring to leaking amniotic fluid or discharge with itching or smell. The categorisation of danger signs was based on the WHO handbook for health care providers and evidence regarding knowledge of danger signs from Tanzania and Madagascar [31–33].

The selection of questions regarding male involvement was based on a literature review of relevant items that reflect male involvement during pregnancy and childbirth [20, 34, 35]. Different items were included: decision making regarding maternal health care issues, financial support for ANC and delivery, and male attendance at ANC consultations. Questions regarding decision making about ANC and delivery (see Table 3) only allowed for one response option; respondents were asked to select the final decision maker. Questions regarding financial support (see Table 3) about ANC and delivery were multiple option questions.

### Data collection

A team of 21 local field workers received a five-day training on ethical issues and data collection procedures, terminology used in the questionnaire and correct translation to the local language (Changana). The team of fieldworkers went from door to door, interviewing all eligible members of the selected households included in the cohort with an electronic questionnaire using tablets. Before the start of the interview, all participants received information regarding the content and objective of the questionnaire, after which written consent was obtained. The questionnaire took on average 30 to 60 min. Inclusion criteria in the cohort study included: speaking Changana or Portuguese, being in a relationship and being between 15 and 49 years old.

### Ethical issues

Ethical approval was obtained from the National Health Bioethics Committee of Mozambique ((187/CNBS/15), Health Bioethics Committee of Universidade Eduardo Mondlane and Hospital Central de Maputo (CIBS UEM&HCM/0008–17) and the Bioethics Committee of Ghent University Hospital (EC/2018/1319).

### Data analysis

All data was analyzed using the statistical software package R. During data cleaning two data entries were deleted because the same participant was interviewed twice, resulting in a final dataset of 775 participants. The χ^2^ test was used for comparing sociodemographic characteristics by sex. The x^2^ test was also used for assessing a relationship between sex and a higher maternal health knowledge (dangers signs and content of ANC), together with confidence intervals for proportions (see Fig. 1 and Fig. 2). The Fisher exact test was computed for cell counts < 5. Descriptive statistics were used for exploring male presence at ANC, decision making and financial support during pregnancy and delivery. Only men and women who experienced a pregnancy in the last 5 years were included in the analysis regarding decision making, financial support and male participation at ANC to control for recall bias.

Within the group of participants that experienced a pregnancy in the last 5 years a subset was selected of couples that were linked based on the question “who is your partner/husband” and year of the last pregnancy. This subset was created for examining level of inter-rater agreement with regard to male presence at ANC, decision making and financial support during pregnancy and delivery. For decision making and financial support answers were categorised under “Man/Woman/Couple together/Others”. Percent agreement was calculated by giving 0 if the man and woman of the same couple had conflicting results (eg the man says he was the final decision maker while the woman says they decided together) and 1 if they had corresponding results (eg the man says the woman was the final decision maker and the woman also says she made the final decision). Cohen’s Kappa was also calculated to examine inter-rater reliability between man and women of the same couple, as it is recommended to use both percent agreement and Cohen’s Kappa in health care studies [36].

A total score of knowledge of danger signs (ranging from 1 to 10) was calculated for all participants in the study by making the sum of danger signs listed by the participants. The Mann-Whitney U Test was used to compare the knowledge score of danger signs between men and women. We examined predictors of knowledge of danger signs for men and women that experienced a pregnancy in the last 5 years by building a binomial logistic regression model. Poor knowledge was defined as knowing less than two danger signs, this cut off value was based on the average number of danger signs respondents knew in this study (=2) and cut off values used in other studies about danger signs during pregnancy [37, 38]. Predictors included education, age, marital status, place of delivery of last child, number of antenatal care consultations during the last pregnancy, if they discussed antenatal care with their partner, male presence during ANC at the last pregnancy and number of living children. The Akaike information criterion (AIC) was used for model selection [39, 40]. *P*-values of less than 0.05 were considered to have significant association between the outcome and the explanatory variables, and P-values of less than 0.1 were considered borderline significant.

## Results

### Sociodemographic characteristics

The study involved 775 participants between the ages of 18 and 54 years, 347 men and 428 women. Four percent (33/808) of the approached participants refused to participate because of time constraints or not being interested. The mean age for men was 36 (ranging from 21 to 54) and for women 32 (ranging from 18 to 53). A total of 491 (63.35%) participants were living in Manhiça district and 284 (36.65%) in Marracuene district. Eight percent of women were in the youngest age category (18–21 years old) and only 1% of men. One in 20 men had followed higher education studies, while only 1 in 100 women had (see Table 2). All participants that did not obtain higher education (*n* = 755) were asked about the reason. The most prevalent reasons were pregnancy and financial reasons. A quarter (26.17%) of women had to stop their studies because of a pregnancy compared to 2% of men. About 69% of men had to stop their studies because of financial reasons, among women this was only 44%. A quarter (26.40%) of women were working in agriculture while only 3% of men had this source of income. More men (39%) were working in the private sector compared to women (5%). Overall women were less educated, younger and more often engaged in domestic work than men (see Table 2).

**Table 2:** Sociodemographic characteristics of the study participants

Sex	Men ***N*** = 347		Women ***N*** = 428		X^**2**^ test	***P***-Value
**Educational level**	**n**	**%**	**n**	**%**	X^2^ = 22.26 (d.f. = 5)	*p* < 0.001
No education	35	10.09	73	17.06		
Primary school (at least 1 year but not finished)	86	24.78	124	28.97		
Primary school (finished)	107	30.84	101	23.60		
Secondary school (at least 1 year but not finished)	81	23.34	104	24.40		
Secondary school (finished)	22	6.34	22	5.14		
Higher education	16	4.61	4	0.93		
**Marital Status**					X^2^ = 6.26 (d.f. = 4)	*p* = 0.18
Single	18	5.19	16	3.74		
Monogamous relationship/Married	292	84.15	362	84.58		
Polygamous relationship/Married	7	2.02	6	1.40		
Divorced/Separated	29	8.36	35	8.18		
Widow	1	0.29	9	2.10		
**Religion**					X^2^ = 6.74 (d.f. = 6)	*p* = 0.35
Catholic	34	9.80	37	8.64		
Islam	8	2.31	10	2.34		
Zione	85	24.50	129	30.14		
Protestant	171	49.28	195	45.56		
Independent Christian church	37	10.66	49	11.45		
No religion	12	3.46	7	1.64		
Others	0	0	1	0.23		
Age**						
18–21	4	1.15	34	7.94	X^2^ = 46.94 (d.f. = 3)	*p* < 0.001
> 21–25	34	9.80	75	17.52		
> 25–35	136	39.19	190	44.39		
> 35	173	49.86	129	30.14		
Occupation**					X^2^ = 365.23 (d.f. = 10)	*p* < 0.001
Public sector (exc. Agriculture)	27	7.78	8	1.87		
Private sector (exc. Agriculture)	135	38.90	22	5.14		
Own business	102	29.39	70	16.36		
Agriculture (commercialized)	8	2.31	14	3.27		
Agriculture (own usage)	10	2.88	113	26.40		
Housekeeper	8	2.31	14	3.27		
Student	1	0.29	6	1.40		
Seasonal worker	18	5.19	2	0.47		
Unemployed	9	2.59	7	1.64		
Homemaker/housewife	0	0	159	37.15		
Others	29	8.36	13	3.04		

Levels of significance:. = *p* < 0.1; * = *p* < 0.05; ** = *p* < 0.01

### Maternal health characteristics

Ninety-nine percent (*n* = 425) of the women had ever been pregnant and 98.27% (*n* = 341) of the men had got a partner pregnant. More than one in three (*n* = 261) of the reported pregnancies were unplanned and 55.94% (*n* = 146) of those unplanned pregnancies were wanted. One hundred forty (18.06%) of the people interviewed had experienced one abortion (spontaneous or induced) with their last partner, 40 (5.16%) two abortions and 7 (0.90%) three abortions or more. A total of 724 respondents (94.51%) had their last child delivered in a health facility, 26 at home, 11 on the road, one in church and three male respondents answered they did not know. For 678 (88.51%) respondents their last child was born by normal vaginal delivery, 60 (7.83%) by vaginal delivery with complications, 26 (3.39%) by caesarean section and two (0.26%) respondents did not know.

### Male involvement in maternal health

Seventy-three percent (*n* = 564) of the participants experienced a pregnancy in the last 5 years, 253 men and 311 women. These participants were asked about decision making, financial support and male attendance during their last pregnancy and delivery. Three percent (*n* = 8) of men said their wives never went to ANC. Of the 245 men whose wives attended ANC, 38.31% (*n* = 95) said they had accompanied her to ANC at least once. Of the women who had been pregnant in the last 5 years, 3.91% (*n* = 13) said they never went to ANC and 30.17% (*n* = 91) of those who went to ANC said their husband had accompanied them at least once. More than three in four women or 77.17% (*n* = 240) would like to have their husband present and 85.26% (*n* = 214) of men would like to be present during ANC.

Almost half of the women (47.88%) said they were the final decision makers regarding going to ANC but only a quarter of men (24.70%) said their wife made the final decision. Only 14.01% of the women said the male partner took the final decision, while 26.29% of men said they were the final decision makers (see Table 3). A majority of women said financial support for ANC came from their partner (79.80%) while only 51% of men said they were providing financial support (see Table 3). Between 0 and 2.3% of respondents (some variation according to sex and topic) stated the parents or parents-in-law were the final decision makers in maternal health care issues. Financial support for antenatal care and delivery from parents and parents-in-law was also minimal, ranging between 0 and 3%.

**Table 3:** Decision making and financial support during pregnancy and delivery among participants experiencing a pregnancy in the last 5 years

Sex	Men (***N*** = 253)		Women (***N*** = 311)	
**Decisions concerning ANC are taken by**	n	%	n	%
Man	66	26.09	149	47.91
Parents-in-law	1	0.40	3	0.96
Other children	0	0.00	0	0.00
Parents	0	0.00	4	1.29
Woman	63	24.90	42	13.50
Couple together	116	45.85	94	30.23
Siblings	0	0.00	0	0.00
Others	7	2.77	19	6.11
**Financial support for ANC (transport and other costs) comes from**^a^				
Man	128	50.59	249	80.06
Parents-in-law	3	1.19	3	0.96
Other children	0	0.00	0	0.00
Parents	2	0.79	5	1.61
Woman	94	37.15	17	5.47
Couple together	26	10.28	30	9.65
Siblings	0	0.00	0	0.00
Nobody	2	0.79	6	1.93
Others	1	0.40	1	0.32
**Decision about place of birth is taken by**				
Man	52	20.55	46	14.79
Parents-in-law	5	1.98	6	1.93
Other children	0	0.00	0	0.00
Parents	2	0.79	7	2.25
Woman	57	22.53	128	41.16
Couple together	124	49.01	98	31.51
Siblings	1	0.40	1	0.32
Others	12	4.74	25	8.04
**Savings during pregnancy for the delivery are done by**^a^				
Man	117	46.25	199	63.99
Parents-in-law	3	1.19	9	2.89
Other children	0	0.00	0	0.00
Parents	4	1.58	9	2.89
Woman	85	33.60	40	12.86
Couple together	39	15.42	46	14.79
Siblings	1	0.40	1	0.32
Nobody	8	3.16	10	3.22
Others	1	0.40	1	0.32

^a^More than one response possible

Among women, 41.04% said they took the final decision about the place of birth, while 20.32% of men said their wife was the final decision maker. One third of women (31.60%) said it was a joint decision with the partner, while half of the men (49.40%) said it was a joint decision (see Table 3).

### Agreement between men and women

Within the group of participants that experienced a pregnancy in the last 5 years, 164 couples (in which both men and women were interviewed) could be identified. We examined the level of agreement (see methods for calculation method of “agreement”) between men and women within this group regarding male presence at ANC during the last pregnancy, decision making and financial support for pregnancy and delivery (see Table 4). We found the highest level of agreement (both percentage of agreement and Cohen’s Kappa) for male presence at ANC, but still the K value was below the often considered acceptable threshold of 0.41 [41]. Overall, we observed a low agreement in what men and women responded. For savings during pregnancy the *P*-value was below 0.05, which means there was no significant association between the responses of men and women (of the same couple).

**Table 4:** Inter rater reliability by percentage of agreement and Cohen’s Kappa among couples

	Percentage Agreement		Cohen’s Kappa	
	n	%	K	***p***-value
Male presence at ANC	107	65.24	0.242	0.0017
Person who takes final decisions concerning ANC	60	36.58	0.095	0.0414
Person who financial support for ANC comes from (transport and other costs)	84	51.21	0.105	0.0085
Person who takes final decision about place of birth	64	39.02	0.124	0.0072
Person who makes savings during pregnancy for the delivery	65	39.63	0.037	0.43

### Maternal health knowledge: ANC content and danger signs

All participants were asked if they knew what happens during an antenatal care consultation and to list what happens during an ANC consultation. 44.09% percent of men and 8.88% of women did not know what happens during ANC. In Fig. 1 an overview of the listed items can be found by sex (with all participants as denominator). There was a significant difference between men and women regarding knowledge about what happens in ANC for almost all listed items: monitoring blood pressure (x^2^ = 10.61178, d.f. = 1, *p* = 0.001), monitoring growth of the baby (x^2^ = 41.83313, d.f. = 1, *p* < 0.001), monitoring weight of the mother (x^2^ = 49.45005, d.f. = 1, *p* < 0.001), providing health information (x^2^ = 46.25236, d.f. = 1, *p* < 0.001), testing the foetal heart rate (x^2^ = 32.06, d.f. = 1, *p* < 0.001) and HIV testing (x^2^ = 69.91854, d.f. = 1, *p* < 0.001). Only regarding family planning counselling (x^2^ = 0.003, d.f. = 1, *p* = 0.95) and other testing (x^2^ = 0.07 d.f. = 1, *p* = 0.80) there was no difference between men and women, but cell counts were very low. Under the category “other testing and treatment”, malaria testing and prevention was most commonly cited and two women also specifically mentioned receiving a bednet.

Participants were asked if they knew any danger signs during pregnancy and to list them. The percentage of men and women that knew certain danger signs can be found in Fig. 2. One danger sign, fitting or convulsions, was not mentioned by any participant. Knowledge of danger signs did not significantly differ between men and women: swollen feet/hands/face (x^2^ = 0.51, d.f. = 1, *p* = 0.47), extreme weakness (x^2^ = 0.17, d.f. =1, *p* = 0.68), painful urination or abnormal vaginal discharge (x^2^ = 0.00, d.f. = 1, *p* = 0.95), strong epigastric pain (x^2^ = 1.17, d.f. = 1, *p* = 0.28), less fetal movements (x^2^ = 0.01, d.f. = 1, *p* = 0.93), fever (x^2^ = 0.95, d.f. = 1, *p* = 0.33), headache or visual problems (x^2^ = 0.09, d.f. = 1, *p* = 0.76) and bleeding (x^2^ = 1.45, d.f. = 1, *p* = 0.23). The average number of danger signs respondents knew was 2.05 (2.00 for men and 2.08 for women), with the difference between men and women not being significant (U = 71,130, *p*-value = 0.294).

Predictors of knowledge of danger signs were examined by building a binomial regression model for men and women separately, with only men and women being pregnant in the last 5 years included. Communication with the partner about ANC was a significant predictor for increased knowledge of danger signs for both men and women (see Tables 5 & 6). We also found that women that did not know how many ANC visits they made in their last pregnancy had lower knowledge about danger signs during pregnancy (see Table 6). For women the number of children was a borderline significant predictor, women with more children had lower knowledge of danger signs. Education, age, marital status, place of last delivery and male attendance during ANC were not significant predictors for knowledge of danger signs.

**Table 5:** Predictors of knowledge of danger signs of men with their coefficients of the binomial regression model

Variables	Poor knowledge of danger signs (0 or 1)	Some Knowledge (2 or more)	Beta coefficient	***P***-Value
	% (n)	% (n)		
**Education**				
No	31.58(6)	68.42(13)	REF	REF
Primary Level	39.02(80)	60.98(125)	−0.32	0.57
Secondary Level	43.75(7)	56.25(9)	−0.31	0.68
Higher	23.08(3)	76.92(10)	−0.24	0.29
**Age**				
18–21	33.33(3)	66.67(6)	REF	REF
21–25	44.44(12)	55.56(15)	−1.02	0.23
25–35	38.94(44)	61.06(69)	−0.55	0.48
> 35	35.58(37)	64.42(67)	−0.63	0.43
**Marital Status**				
Single	37.14(13)	62.86(22)	REF	REF
In relationship	38.07(83)	61.93(135)	−0.13	0.76
**Place of last delivery**				
Hospital	37.92(91)	62.08(149)	REF	REF
At home	38.46(5)	61.54(8)	0.06	0.92
**Number of ANC visits**				
0 ANCs	37.50(3)	62.50(5)	REF	REF
< 4 ANCs	28.57(10)	71.43(25)	0.24	0.78
> =4 ANCs	29.36(32)	70.64(77)	0.13	0.87
Don’t know	50.50(51)	49.50(50)	−0.58	0.47
Communication regarding ANC*				
No	53.09(43)	46.91(38)	REF	REF
Yes	30.81(53)	69.19(119)	0.73	0.01
**Male attendance last pregnancy**				
No	43.23(67)	56.77(88)	REF	REF
Yes	29.59(29)	70.41(69)	0.32	0.29
**Number of children alive**				
0–2	32.35(11)	67.65(23)	REF	REF
3–5	38.51(62)	61.49(99)	−0.30	0.49
> 5	39.66(23)	60.34(35)	− 0.39	0.43

Levels of significance:. = *p* < 0.1; * = *p* < 0.05; ** = *p* < 0.01

**Table 6:** Predictors of knowledge of danger signs of women with their coefficients of the binomial regression model

Variables	Poor knowledge of danger signs (0 or 1)	Some Knowledge (2 or more)	Beta coefficient	*P*-Value
	% (n)	% (n)		
**Education**				
No	31.43(11)	68.57(24)	REF	REF
Primary Level	28.46(72)	71.54(181)	−0.07	0.86
Secondary Level	30.00(6)	70.00(14)	−0.27	0.65
Higher	66.66(2)	33.33(1)	−1.07	0.46
**Age**				
18–21	29.55(13)	70.45(31)	REF	REF
21–25	29.51(18)	70.49(43)	0.01	0.99
25–35	29.61(45)	70.39(107)	0.02	0.98
> 35	27.78(15)	72.22(39)	0.24	0.62
**Marital Status**				
Not in relationship	28.89(13)	71.11(32)	REF	REF
In relationship	29.32(78)	70.68(188)	−0.14	0.73
**Place of last delivery**				
Hospital	29.35(86)	70.65(207)	REF	REF
At home	27.78(5)	72.22(13)	0.32	0.59
Number of ANC visits*				
0 ANCs	53.85(7)	46.15(66	REF	REF
< 4 ANCs	32.08(17)	67.92(36)	0.87	0.18
> =4 ANCs.	28.38(63)	71.62(159)	1.03	0.08
Don’t know*	17.39(4)	82.61(19)	1.73	0.03
Communication regarding ANC*				
No	40.00(30)	60.00(45)	REF	REF
Yes*	25.85(61)	74.15(175)	0.68	0.03
**Male attendance last pregnancy**				
No	31.82(70)	68.18(150)	REF	REF
Yes	23.08(21)	76.92(70)	0.33	0.32
**Number of children alive**				
0–2	17.07(7)	82.93(34)	REF	REF
3–5.	30.57(59)	69.43(134)	−0.88	0.06
> 5.	32.47(25)	67.53(52)	−0.95	0.06

Levels of significance:. = *p* < 0.1; * = *p* < 0.05; ** = *p* < 0.01

## Discussion

This study sheds light on men’s and women’s knowledge, decisions and behaviour related to some critical maternal health care issues in Mozambique. It is important to note that relatively few maternal health care studies collect data from male partners directly [20], while our study shows men and women often have different views on decision making, financial support and presence at antenatal care consultations. As a consequence, data on male attitudes, knowledge or behaviour in maternal health care issues deriving from women only should be interpreted with caution.

The sociodemographic data of our study population showed that women are often younger at the first pregnancy compared to men, more often have to stop their education because of pregnancy and are more often engaged in informal jobs. This is in line with other studies in Mozambique demonstrating that women are still disadvantaged in terms of education, employment and income [16, 42]. As a result women often rely on their partners for financial support in their daily life [16]. However, the rapid urbanization in southern Mozambique may also lead to an enhanced socio-economic space for women, as women seem to have more socio economic power and possibilities in cities [42]. Our study demonstrates that decisions regarding ANC and delivery are mostly taken by women, followed by the couple jointly. Although women might take the lead in these decisions, the majority of them report that they rely on their partner for providing financial support regarding antenatal care and delivery (80 and 64% respectively). In contrast to some qualitative studies in the region [16, 43] the role of mothers–in-law seems relatively small in terms of decision making and financial support.

Our study showed a high level of male participation at ANC, much higher than the figures reported in other studies. This might be related to participants’ understanding of “going with your wife to ANC”. A qualitative study about male involvement in southern Mozambique showed that men and women often consider male accompaniment to include going with your partner until the gate of the clinic [44] and similarly an Ethiopian study reported different understandings of male accompaniment at ANC [18]. Moreover, only 64% of the couples gave the same answer to this question, indicating that men and women may have different interpretations. Socially desirable answers might have also contributed to this result.

Both men and women show a high willingness for male participation at ANC in our study, but some persistent barriers and potential negative consequences might deter their actual presence at ANC. Research demonstrates that health care centres in rural Mozambique already struggle to offer high quality ANC [7] and receiving a high number of male partners will create additional challenges. Moreover, couples attending ANC are often assumed to be HIV positive by the community, with the associated stigma [44, 45]. Finally, and importantly, a qualitative study in the region emphasized women are often treated in an inferior manner to men by health care providers when both are present in the consultation [44]. To tackle these barriers to male involvement in maternal health while also promoting gender equality, tailored programs at different levels will be needed.

Overall we noted that men often believed they played a more important role in maternal health care issues (regarding decision making and participation in ANC) than reported by their female counterparts. Furthermore, we observed that men and women each believed that it was their partner’s responsibility to prepare savings for the delivery and organise financial support for ANC (which might indicate no one was actually assuming responsibility for this). For savings during pregnancy for example, the majority of women said the partner kept money aside (64%) while only 46% percent of men said they did so.

Universal access to ANC offers an opportunity to encourage women to deliver within a health facility, and can function as an entry point for health care for the whole family [46]. Notably, almost all women (97%) in our study had accessed antenatal care in their last pregnancy, which is a promising result in terms of ANC coverage. Our study revealed which components of ANC are most commonly known by women and their partners. Health promotion (such as nutritional advice), monitoring of the growth of the baby (by measuring fundus height) and HIV testing were the most commonly known ANC interventions among women (> 50%) and their partners (> 25%). Nevertheless, some crucial interventions were much less known such as hypertension screening and malaria prevention and treatment. Our study thereby confirms the results of other studies in Mozambique showing that blood pressure screening is often neglected by providers or impossible due to lack of equipment [6, 7]. As we expected, women knew more about the content of ANC compared to men. This supports the view that ANC is often considered as women’s business [47] and men are much less exposed to and familiar with ANC.

In contrast with knowledge of ANC content, the knowledge of danger signs during pregnancy did not differ between men and women. Moreover, knowledge was very low and did not correlate with presence at ANC (either of the woman or her partner). Low knowledge of danger signs among women was also reported in studies in Madagascar, Tanzania and Ethiopia and often indicates overall poor quality of antenatal care [33, 38, 48]. Assessment of knowledge of danger signs among men is rarely carried out within maternal health care research, although it is well known that they play a major role in the referral of pregnant women in case of emergencies. One study in Tanzania indicated low knowledge amongst men, but this was not compared with women’s knowledge in the same setting [38]. We believe our study is the first to examine differences between men and women regarding knowledge of danger signs in Sub-Saharan Africa. Since principally women are targeted in maternal health care programs we would have expected a higher level of knowledge among women [49] but this was not the case. The low level of knowledge of danger signs among both men and women suggests that counselling on danger signs during ANC is not routinely carried out or does not increase women’s knowledge. DHS data from 2015 in Mozambique demonstrated only 39% of women were counselled on danger signs during ANC, and our results suggest this proportion has not significantly changed in 2017. Given that knowledge of danger signs is an essential step in the timely referral of pregnant women in case of emergencies (often called the first delay), this is definitely an aspect of ANC that needs more attention. The fact that men will often act as gatekeepers to safe maternity care should be taken into account when designing education, communication and information programs for improving maternal and neonatal health outcomes. The inclusion of men in maternal health care programs is still often neglected, while the International Conference on Population and Development (ICPD) already argued in 1994 that special efforts should be made to emphasize on men’s shared responsibility and active involvement in maternal and child health [50].

Communication about antenatal care within the couple was a significant predictor for better knowledge of danger signs among both men and women. Several possible mechanisms might explain the link between communication and knowledge. First, awareness of danger signs may have provided the couple with the opportunity to start conversations about the pregnancy and content of ANC. Second, the fact that they communicate about the content of ANC might have increased their knowledge. While the positive effect of couple communication on consistent family planning usage has been demonstrated [51, 52], this has been much less studied in terms of ANC attendance, maternal health care knowledge and skilled birth attendance. In light of the findings of our study we suggest that male involvement programs also keep track of “soft” indicators of male involvement such as couple communication, interest of the partner and shared decision making to evaluate their programs, instead of focusing on male participation in ANC visits as the main core indicator especially because the latter is often interpreted differently by different actors (such as the woman, partner and health care provider).

Our study has several limitations worth noting. The study was nested within the third annual round of a cohort study. Therefore all participants had already reached the age of 18 years old by the time we included them in this study. According to DHS data of 2015, 44% of Mozambican girls had been pregnant at least once by the age of 17 [15]. Consequently our study is biased by only gathering data from participants 18 years old and above. Another shortcoming of our study was the absence of questions regarding income or financial stress in our questionnaire, which means we could not assess a potential association between economic status and knowledge of danger signs. Especially for the association between having more children and lower knowledge of danger signs we suspect that economic status might have been a confounding factor, but we were unable to verify this hypothesis. Another methodological limitation is our measurement of knowledge of danger signs. In line with other studies we took knowledge of less than 2 danger signs as cut-off point, but this is an arbitrary and unnuanced approach. Future studies should explore the design of a more refined and validated instrument for measuring knowledge of danger signs during pregnancy and childbirth.

Lastly, it should be taken into account that this study was conducted in Manhica and Marracuene, two districts close to the capital, a region that is advantaged in terms of economic resources compared to the rest of the country. In addition, Northern Mozambique has a matrilineal marital, kinship and inheritance system while southern Mozambique has a patrilineal system [53]. Taking into account these regional differences, our findings cannot be generalized to other parts of the country.

## Conclusions

This study shows that men play an important role in decision making and financial support in maternal health care issues in southern Mozambique. The role of parents, parents-in-law and neighbors was rather small. Couples often had different opinions on who took the decisions, who provided financial support and male participation in antenatal care. This finding of disagreement within couples is interesting as many maternal health care studies rely on women’s reports only for assessing the role of the male partner [20]. We recommend that future maternal health care research should collect more data from men directly and assess male involvement more broadly than presence at ANC. This study showed a high willingness for more male participation at ANC by both men and women, which should encourage policy-makers to invest in multilevel tailored interventions tackling current barriers. Improving maternal health care knowledge in the community can improve maternal health outcomes and should go hand in hand with the promotion of male involvement and gender equality.

## Supplementary information

**Additional file 1.** Questionnaire. Description: The questionnaire used for the current study in Portuguese and English.

## Data Availability

For guaranteeing anonymity and confidentiality of our participants the dataset used and analyzed during the current study are only available from the corresponding author upon reasonable request.

## Abbreviations

AIC: Akaike information criterion
ANC: Antenatal Care
ICPD: International Conference on Population and Development
LMIC: Low and Middle Income Countries
MCH: Mother and Child Health
MDGs: Millennium Development Goals
MMR: Maternal Mortality Ratio
MoH: Ministry of Health
SDGs: Sustainable Development Goals
UEM: Universidade Eduardo Mondlane
WHO: World Health Organization
