# Supplementary material for: A cross-sectional study of the role of men and the knowledge of danger signs during pregnancy in southern Mozambique
Source: BMC Pregnancy Childbirth. 2020 Sep 29;20:572. doi: 10.1186/s12884-020-03265-4 (PMC7526108; doi:10.1186/s12884-020-03265-4)
Supplement: Supplementary file 1 — Additional file 1. Questionnaire. Description: The questionnaire used for the current study in Portuguese and English. [file 12884_2020_3265_MOESM1_ESM.docx]

**ENGLISH VERSION**

**H. Involvement of men in Pregnancy and Childbirth**

**(Only for women who have been pregnant at least once and men whose partners have been pregnant at least once)**

H0. Have you or your partner been pregnant at least once?

(1) Yes

(2) No → (go to H12)

H1. When were you / your partner last pregnant?

Year: ____ (4 digits)

H2.How many antenatal consultations did you (or your partner / wife) have during the last pregnancy?

(1) None

(2) 1

(3) 2

(4) 3

(5) 4

(6) 5 or more

(7) Don't know

H3. Who took the final decision about going or not going to antenatal care consultations? (Only one answer option)

(1) Me

(2) Mother-in-law

(3 Children

(4) Mother / Father

(5) Husband / Wife / Partner

(6) Together (Me and my partner)

(7) Brothers or sisters (-in-law)

(8) Others, Please specify: …………………………………………

D20.3 What was the place of the last birth?

(1) At the health center / hospital

(2) At home

(3) On the way to the health center / hospital

(4) Other, Please specify: …………………………………………

H4. Who took the final decision about the place of delivery? (Only one answer option)

(1) Me

(2) Mother-in-law

(3 Children

(4) Mother / Father

(5) Husband / Wife / Partner

(6) Together (Me and my partner)

(7) Brothers or sisters (-in-law)

(8) Others, Please specify: …………………………………………

D20.4. If you did not deliver at the health center / hospital, why? (Multiple response options)

(1) Lack of transportation

(2) Hospital was too far

(3) Labor started at night

(4) Did not know the exact date of the child's birth

(5) I was home alone

(6) I preferred to have the child at home

(7) Other, Please specify: …………………………………………

D20.5. How was the last delivery?

(1) Normal without complications

(2) Caesarean section

(3) With complications

(4) Don't know

H5. For women only: Has your husband / partner ever accompanied you to antenatal care consultations at the health facility?

(1) Yes

(2) No

H6. For men only: Have you ever accompanied your partner / wife to antenatal consultations at the health facility?

(1) Yes

(2) No

H7. For women only: Would you like to be accompanied by your partner / husband when going for antenatal care consultations?

(1) Yes

(2) No

H8. Only for men: Would you like to accompany your partner / wife to antenatal consultations?

(1) Yes

(2) No

H9. Who provides money for transportation or other expenses when you go to antenatal consultations at the health facility?

(1) Me

(2) Mother-in-law

(3 Children

(4) Mother / Father

(5) Husband / Wife / Partner

(6) Together (Me and my partner)

(7) Brothers or sisters (-in-law)

(8) Others, Please specify: …………………………………………

H10. During pregnancy, who saves money for the expenses that come with the baby's birth?

(1) Me

(2) Mother-in-law

(3 Children

(4) Mother / Father

(5) Husband / Wife / Partner

(6) Together (Me and my partner)

(7) Brothers or sisters (-in-law)

(8) No one

(9) Others, Please specify: …………………………………………

H11. Do you talk with your partner about what happens during antenatal care consultations?

(1) No

(2) Yes

H12. Do you know what happens in antenatal care consultations?

(1) No

(2) Yes

H.13 If Yes, what happens? (Do not read the answer options)

(1) Provide information about the pregnancy and baby

(2) Follow up on the development of the pregnancy and baby

(3) Blood pressure screening

(4) HIV testing

(5) Control of the fetal heartbeat

(6) Weight gain control

(7) Others, Please specify: …………………………………………

H14. Do you know where to go in case of emergency or any particular problem during pregnancy or childbirth?

(1) No

(2) Yes

H15 If yes, where to ___________________

H16. What are the danger signs during pregnancy or severe problems (that are not normal)? (Do not read the options)

(1) Bloodloss through the vagina

(2) Strong headache or blurred vision

(3) Fever

(4) Decreased fetal movements

(5) Strong and persistent pain in the stomach

(6) Persistent vomiting

(7) Burning or painful urination

(8) Itchy or foul-smelling vaginal discharge

(9) Convulsions

(10) Others: Please specify: …………………………………………

**PORTUGUESE VERSION**

**H. Envolvimento do Homem na Gravidez e Parto**

**(Apenas para mulheres que tiveram pelo menos uma gravidez e homens cujas parceiras engravidaram pelo menos uma vez)**

H0. Você ou sua parceira já esteve grávida pelo menos uma vez?

1. Sim
2. Não → (passa para H12**)**

H1. Quando você/sua parceira esteve grávida pela última vez?

Ano:____(4 digitos)

H2.Quantas consultas pré-natais (a sua parceira/actual esposa) fez durante a última gravidez?

(1) Nenhuma

(2) 1

(3) 2

(4) 3

(5) 4

(6) 5 ou mais

(7) Não sabe

H3. De quem foi a decisão final de ir ou não as consultas pré-natais? (Apenas uma única opção de resposta)

(1) Própria

(2) Sogra/Sogro

(3) Filhos

(4) Mãe/Pai

(5) Marido/Esposa/Parceiro/Parceira

(6) Ambos (marido/parceiro e mulher/parceira)

(7) Irmãos/Cunhados

(8) Outros, Especifique : …………………………………………

D20.3 Onde você/sua parceira teve o último parto?

(1) Na unidade sanitária/hospital

(2) Em casa

(3) A caminho da US/hospital

(4) Outro, Especifique: …………………………………………

H4. De quem foi a decisão final sobre o local do parto? (Apenas uma única opção de resposta)

(1) Própria

(2) Sogra/Sogro

(3) Filhos

(4) Mãe/Pai

(5) Marido/Esposa/Parceiro/Parceira

(6) Ambos (marido/parceiro e mulher/parceira)

(7) Irmãos/Cunhados

(8) Outros, Especifique : …………………………………………

D20.4. Se não deu parto na unidade sanitária/hospital, porquê? (Múltiplas opções de resposta)

(1) Falta de transporte

(2) Hospital era distante

(3) Trabalho de parto começou durante a noite

(4) Não sabia da data exacta do nascimento da criança

(5) Estava sozinha em casa

(6) Preferi ter a criança em casa

(7) Outro, Especifique : …………………………………………

D20.5. Como foi o último parto?

(1) Normal s/ complicações

(2) Cesariana

(3) Normal c/ complicações

(4) Não sabe

H5. Apenas para mulheres: O seu esposo/parceiro alguma vez te acompanhou as consultas pré-natais na unidade sanitária?

(1) Sim

(2) Não

H6. Apenas para homens: Você alguma vez acompanhou a sua parceira/esposa as consultas pré-natais na unidade sanitária?

(1) Sim

(2) Não

H7. Apenas para mulheres: Gostaria que o seu companheiro/parceiro a acompanhasse as consultas pré-natais?

(1) Sim

(2) Não

H8. Apenas para homens: Gostaria de acompanhar a sua parceira/esposa as consultas pré-natais?

(1) Sim

(2) Não

H9. Quem lhe dá dinheiro para transporte ou outras despesas quando vai as consultas pré-natais na unidade sanitária?

(1) Próprio/a

(2) Sogra/Sogro

(3) Filhos

(4) Mãe/Pai

(5) Marido/Esposa/Parceiro/Parceira

(6) Ambos (marido/parceiro e mulher/parceira)

(7) Irmãos/Cunhados

(8) Outros: Especifique : …………………………………………

H10. Durante a gravidez quem guarda dinheiro para as despesas que vem com o nascimento do bebê?

(1) Própria/o

(2) Sogra/Sogro

(3) Filhos

(4) Mãe/Pai

(5) Marido/Esposa/Parceiro/Parceira

(6) Ambos (marido e mulher)

(7) Irmãos/Cunhados

(8) Ninguém

(9) Outros, Especifique :…………………………………………

H11. Você conversa com seu parceiro/parceira sobre o que acontece nas consultas pré-natais?

(1) Não

(2) Sim

H12. Você sabe o que acontece nas consultas pré-natais?

(1) Não

(2) Sim

H.13 Se sim, o que acontece? (Não leia as opções de resposta)

(1) Dão informação sobre a gravidez e bebé

(2) Desenvolvimento da gravidez e do bebê

(3) Controle de pressão arterial

(4) Teste de HIV

(5) Teste de batimento cardíaco fetal

(6) Controle de ganho de peso

(7) Outros, Especifique: …………………………………………

H14. Você sabe para onde deve ir em caso de algum problema ou emergência durante a gravidez ou parto?

(1) Não

(2) Sim

H15 Se sim, para onde ___________________

H16. Quais são os sinais de alerta ou problemas que podem ocorrer durante a gravidez que não são normais? (Não leia as opções de resposta)

(1) Perda de sangue pela vagina

(2) Dor de cabeça forte ou visão turva

(3) Febre

(4) Diminuição dos movimentos fetais

(5) Dor forte e persistente no estômago

(6) Vômito persistente

(7) Ardência ou dor ao urinar

(8) Corrimento vaginal com coceira ou mau cheiro

(9) Convulsões

(10) Outros: Especifique: …………………………………………
